# Cognitive-Behavioral Therapy for Postbariatric Surgery Patients With Mental Disorders: A Pilot Study

**DOI:** 10.3389/fpsyt.2020.00014

**Published:** 2020-02-12

**Authors:** Almut Rudolph, Anja Hilbert

**Affiliations:** ^1^Clinical Psychology and Psychotherapy, Department of Psychology, Faculty of Life Sciences, University of Leipzig, Leipzig, Germany; ^2^Integrated Research and Treatment Center Adiposity Diseases, Departments of Medical Psychology and Medical Sociology and Psychosomatic Medicine and Psychotherapy, Leipzig University Medical Center, Leipzig, Germany

**Keywords:** cognitive-behavioral therapy, psychotherapy, bariatric surgery, binge-eating disorder, major depressive disorder

## Abstract

**Background:**

Binge-eating disorder (BED) and major depressive disorder (MDD) following bariatric surgery are significant predictors for less post-operative weight loss and/or weight regain, however, cognitive-behavioral therapy (CBT) addressing these disorders following surgery has not been investigated so far.

**Objective:**

This study examined feasibility of a short-term CBT based on evidence-based manuals for BED and MDD that were adapted to patients following bariatric surgery, and investigated its effectiveness in improving weight loss outcome, psychopathology, and psychosocial functioning.

**Materials and Methods:**

In an uncontrolled proof-of-concept study, the CBT manual was piloted in N = 7 patients who had undergone roux-en-Y gastric bypass surgery at least 6 months before. Weight loss, eating disorder psychopathology, depressive symptoms, and self-esteem were assessed using clinical interviews and self-report questionnaires at pre-treatment, post-treatment, and in a 3-month follow-up.

**Results:**

A significant reduction of body weight was found as well as medium to large effects in the improvement of eating disorder psychopathology, depressive symptoms, and self-esteem from pre-treatment to post-treatment were found. Most of those changes remained stable during the 3-month follow-up period. Study retention was 71.4%.

**Conclusions:**

Feasibility and effectiveness of CBT were documented for patients with BED or MDD following bariatric surgery. Adaptations of the study procedure for proof-of-efficacy in randomized-controlled studies are discussed.

## Introduction

Bariatric surgery is the only efficacious treatment for patients with severe obesity (body mass index, BMI ≥ 40 kg/m^2^ or ≥ 35 kg/m^2^ with comorbidity), resulting in substantial long-term weight loss up to 20 to 35% of initial body weight and decreased morbidity and mortality (1–4). However, research has shown that 20% of patients experience only minor weight loss 1 year after surgery (5, 6) and 33% of patients experience minor weight loss 10 years after surgery (7). Additionally, up to 50% of patients experience weight regain within 2 years after surgery (8).

Significant predictors for less post-operative weight loss and/or weight regain have been identified with post-operative eating disturbances or disorders (9–13), e.g. binge-eating disorder (BED) or loss of control (LOC) eating as well as major depressive disorder (MDD) in up to 27% (14) and 17% (15) of the patients, respectively. For example, post-operative LOC eating leads to a ≥ 8 lesser BMI loss and weight regain in the long-term (16, 17) and post-operative MDD was associated with ≥ 10% lesser percentage of weight loss in the third year following surgery (15). In addition to insufficient weight loss outcome, patients with post-operative LOC or MDD suffer from increased eating disorder and general psychopathology, and impairments in quality of life (16, 17).

Although recommended in clinical guidelines for bariatric surgery (18–20), post-operative behavioral management is not performed systematically and is not sufficiently based on evidence. Two meta-analyses on randomized-controlled trials (RCTs) documented the positive effect of post-operative behavioral management on post-operative weight loss compared to usual care. However, primarily addressing diet and physical activity in patients unselected for psychopathology, neither study comprehensively addressed post-operative mental disorders diagnosed with clinical interviews (21, 22). To the best of our knowledge, only one uncontrolled study examined a six session telephone-based cognitive-behavioral therapy (CBT) and found patients following bariatric surgery unselected for psychiatric symptoms experiencing significant reductions in depressive and anxious symptoms pre- to post intervention (23, 24).

Despite the clinical relevance of BED and MDD following bariatric surgery, there is no study to date that examined psychological treatment for patients with diagnosed post-operative mental disorders. Therefore, we aimed at piloting a CBT, the most established treatment for patients with BED (25) and MDD (26). The CBT manual comprised evidence-based interventions focusing on BED and MDD adapted to the specific needs of patients following bariatric surgery. In an uncontrolled proof-of-concept study, we examined feasibility (e.g. acceptance, retention) and sought to evaluate the effects of the CBT manual in improving weight loss outcome (e.g. BMI). Furthermore, changes in eating disorder and general psychopathology, as well as psychosocial functioning, were assessed.

## Methods

### Patients, Recruitment, and Study Procedure

Patients (aged ≥ 18 years) were eligible if they had undergone roux-en-Y gastric bypass (RYGB) surgery at least 6 months ago and were post-operatively diagnosed with BED and/or MDD. Patients were excluded when they were diagnosed with psychotic disorder, bipolar disorder, substance-related disorder, current suicidal ideation, and ongoing psychotherapy. The study was approved by the local institutional review board and written informed consent was obtained from all patients prior to study enrollment. Patients were recruited at the obesity outpatient clinic of the Integrated Research and Treatment Center Adiposity Diseases, University of Leipzig Medical Center, Germany. During their regular post-operative follow-up visits, the interdisciplinary team informed all patients about the study. Patients who were interested were screened via telephone interview. Eligible patients were invited to a diagnostic session. Assessments included objective measurements of weight and height, structured clinical interviews, and self-report questionnaires, and were conducted at pre-treatment and post-treatment (in-person visits), and at 3-month follow-up (telephone interviews and posted questionnaires) by a trained and supervised assessor.

### Cognitive-Behavioral Therapy (CBT)

We compiled a modular short-term psychotherapy manual selecting interventions from two evidence-based CBT manuals for BED (27) and MDD (28), in order to meet the needs of patients with post-operative mental disorders. In addition to these established interventions for BED (e.g. self-monitoring of food intake) and/or MDD (e.g. thought records), the POSTBAR manual (Post-operative CBT for bariatric surgery patients) included adaptations focused on nutritional and behavioral recommendations following bariatric surgery (e.g. portion sizes, supplementation). The CBT manual comprised 15 individual face-to-face sessions à 50 min within 5 months. Within the first 3 months, sessions were scheduled weekly, while the last three sessions were scheduled biweekly. The initial treatment phase aimed for motivational enhancement (three sessions), the intensive treatment phase comprised modules on BED and/or MDD (nine sessions), and the final self-management phase focused on relapse prevention (three sessions). All sessions were delivered by a licensed cognitive-behavioral therapist (first author), and were regularly supervised (second author). Therapy adherence was defined as the attendance of at least 12 sessions (29).

### Measures

#### BMI

At pre- and post-treatment, BMI was calculated from measured body weight and height. At 3-month follow-up, body weight was self-reported.

#### Diagnosis of BED and eating disorder psychopathology

Post-operative eating behavior within the last 3 months was assessed using an adapted German version of the semi-structured Eating Disorder Examination interview (EDE) (30). The EDE–Bariatric Surgery Version (EDE-BSV) comprised diagnostic items as well as additional items to assess eating behavior following bariatric surgery (17). According to the fifth edition of the *Diagnostic and Statistical Manual of Mental Disorders* (31), BED is defined through recurrent objective binge-eating episodes (OBEs; eating an objectively large amount of food accompanied by a sense of LOC over eating in the absence of compensatory behaviors). As the patients' ability to consume an objectively large amount of food following bariatric surgery is limited, subjective binge-eating episodes (SBEs; eating a subjectively large amount of food with concomitant LOC) were allowed for full-syndrome diagnosis of BED in addition, in line with previous research (17). Further, subsyndromal BED (i.e. presence of two behavioral indicators or lack of distress) (29), and BED of low frequency/limited duration were diagnosed (i.e. binge-eating episodes less than once per week) (31). Furthermore, the German version of the EDE-Questionnaire (EDE-Q) was administered to assess eating disorder psychopathology during the past 28 days with 22-items answered on a seven-point scale (0 = feature was absent, 6 = feature was present to an extreme degree) (32). A global mean score was computed with higher scores indicating higher levels of global eating disorder psychopathology.

#### Diagnosis of MDD and depressive disorder psychopathology

The Structured Clinical Interview for DSM-5 diagnoses (33) was used to diagnose current MDD. The severity of depressive symptoms during the past two weeks was measured using the nine items of the depression module of the German version of the Patient Health Questionnaire (PHQ-9) (34). The nine items were answered on a four-point scale (0 = not at all, 3 = nearly every day) with a higher sum scores indicated higher levels of depressive symptoms.

#### Quality of life

Obesity-specific quality of life was assessed with the German version of the Impact of Weight on Quality of Life–Lite (35). Patients answered the 31 items on a five-point scale (0 = never true, 4 = always true). Higher sum scores indicated poorer quality of life during the last week.

#### Self-esteem

Overall liking of oneself was measured with the ten items of the German version of the Rosenberg Self-Esteem Scale (36). Items were answered on a four-point scale (0 = strongly disagree, 3 = strongly agree), and higher mean scores indicated higher levels of self-esteem.

#### Feasibility

To assess acceptance, patients answered the questions “How satisfied have you been with the treatment?” and “How helpful was the treatment for your problem?” on ten-point scales (0 = not at all; 10 = very much) at post-treatment.

### Data Analysis

Data analysis was carried out using the Statistical Package for the Social Sciences (SPSS 24.0 for Windows; (37). Descriptive analyses were used to report feasibility and to present patients' characteristics. Inferential analyses were based on all patients applying intent-to-treat analyses using last observation carried forward methods for the two patients who did not finished treatment nor assessments, a patient who did not return the questionnaires at 3-month follow-up and for a patient who received an unplanned revision surgery during treatment (i.e. pre-revisional body weight carried forward for post-treatment and 3-month follow-up). Changes from pre-treatment to post-treatment and 3-month follow-up were analyzed using nonparametric Fisher's exact tests for categorical variables and Friedman tests for continuous variables. For the latter, Wilcoxon signed-rank tests including Bonferroni-Holm corrections were run in order to calculate effect sizes. Effect sizes φ and *r* with ≤ 0.3, ≤ 0.5, and ≥ 0.5 correspond to small, medium, and large effects, respectively (38). Significance was determined at a two-tailed *p* < 0.05.

## Results

### Sample Characteristics

Seven patients were included (four females, M ± SD: age 48.6 ± 10.0 years, BMI 41.85 ± 4.34 kg/m^2^). Two patients were diagnosed with MDD as primary diagnosis and BED as a secondary diagnosis, four patients were diagnosed with BED only, and one patient was diagnosed with MDD only (**Table 1**).

**Table 1:** Patient characteristics and individual changes in clinical variables for pre-treatment, post-treatment, and 3-month follow-up assessments.

Patient characteristics	Patient A				Patient B					Patient C			
Age	60–65				30–35					50–55			
Diagnosis	BED with SBEs; MDD				BED with SBEs (sub); MDD					BED with SBEs			
Months since RYGB	8				7					21			
Weight (kg) before RYGB	166				195					117			
Weight regain in kg after nadir^a^	4				None					6			
Individual changes	Pre-treatment	Post-treatment	3-month follow-up		Pre-treatment	Post-treatment		3-month follow-up		Pre-treatment	Post-treatment		3-month follow-up
Body weight in kg	139.9	129.0	127.0		134.9	123.9		121.2		95.3	93.7		91.6
BMI in kg/m^2^	51.4	47.4	46.7		38.6	35.4		36.2		37.7	37.1		36.2
%EWL	43.0	61.0	64.3		78.2	92.5		96.0		37.7	40.5		44.2
BED/MDD diagnosis^b^	1/1	0/0	0/0		1/1	0/1		1/0		1/0	1/0		0/0
EDE-Q	4.1	2.3	2.5		0.9	0		0		2.6	3.5		3.8
PHQ-9	17	11	12		12	9		9		7	7		11
IWQOL	101	92	92		3	3		3		38	29		35
RSES	1.8	2.3	2.5		3.1	3.7		3.7		3.1	3.1		3.4
**Patient characteristics**	**Patient D**				**Patient E**					**Patient F^c^**		**Patient G^c^**	
Age	46–50				36–40					46–50		56–60	
Diagnoses	BED with SBEs (sub)				MDD					BED (sub)		BED	
Months since RYGB	63				19					25		78	
Weight in kg before RYGB	155				180					180		146	
Weight regain in kg after nadir^a^	20				None					4		38	
Individual changes	Pre-treatment	Post-treatment		3-month follow-up	Pre-treatment		Post-treatment		3-month follow-up	Pre-treatment		Pre-treatment	
Body weight in kg	122.0	120.0		120.0	139.6		131.0		130.0	128.5		115.9	
BMI in kg/m^2^	38.9	38.3		38.3	43.6		40.9		40.6	40.6		42.1	
%EWL	46.6	49.4		49.4	56.2		68.2		69.5	72.2		49.1	
BED/MDD diagnosis^b^	1/0	0/0		1/0	0/1		0/0		0/0	1/0		1/0	
EDE-Q	4.0	2.7		2.6	2.8		3.6		2.5	3.1		4.4	
PHQ-9	12	0		6	17		6		5	14		16	
IWQOL	83	47		36	41		70		70	29		85	
RSES	2.2	2.9		3.5	1.7		2.5		2.6	2.8		1.3	

^a^Nadir = lowest weight after bariatric surgery. ^b^Dichotomous: 0 = no, 1 = yes. ^c^Patient did not complete treatment and diagnostic assessments, for all further analysis, intention-to-treat analysis with last observation carried forward method was applied. BED, binge-eating disorder; BMI, body mass index; EDE-Q, Eating Disorder Examination-Questionnaire (0–6); IWQOL, Impact of Weight on Quality of Life (0–4); MDD, major depressive disorder; PHQ, Patient Health Questionnaire (0–3); RSES = Rosenberg Self-Esteem Scale (0–3); SBE, subjective binge-eating episodes; sub, subsyndromal; %EWL, percentage of excess weight loss.

### Feasibility

During 3 months of recruitment, 98 patients with at least 6 months since RYGB surgery were scheduled for regular post-operative follow-up visits. Overall, 16 patients were identified as in need of psychological treatment and were screened by the study team. From those patients, nine were excluded for organizational reasons (i.e. lack of time; N = 3), travel distance (N = 3), and lack of psychopathology (N = 3). Seven patients completed treatment and follow-up assessments, however, two patients (F, G) dropped out before end of treatment (session 10 and session 6, respectively), resulting in a retention rate of 71.4%. Individual treatment sessions were scheduled according to the study protocol. Patients indicated good acceptance of treatment (satisfaction: 7.2 ± 4.2; helpfulness: 7.4 ± 3.0).

### Pre- to Post-Treatment and Follow-Up Changes

Individual and overall changes in clinical variables are presented in **Tables 1** and **2**. For weight loss, we found significant changes: with large effects, weight loss increased from pre- to post-treatment as well from pre-treatment to 3-month follow-up.

**Table 2:** Differences in clinical variables for pre-treatment, post-treatment, and 3-month follow-up assessments.

	*M* (*SD*)			*χ*^2^(2)	*Z* (*r*)		
	Pre-treatment	Post-treatment	3-month follow-up		Pre-treatment vs. post-treatment	Pre-treatment vs. 3-month follow-up	Post-treatment vs. 3-month follow-up
Body weight in kg	125.2 (15.9)	120.3 (12.9)	119.2 (13.2)	9.6**	−2.0* (0.8)	−2.0* (0.8)	−1.8^†^ (0.7)
BMI in kg/m^2^	41.8 (4.7)	40.2 (3.9)	39.9 (4.0)	9.6**	−2.0* (0.8)	−2.0* (0.8)	−1.8^†^ (0.7)
%EWL	54.7 (15.2)	61.8 (17.6)	63.5 (18.0)	9.6**	−2.0* (0.8)	−2.0* (0.8)	−1.8^†^ (0.7)
EDE-Q	3.0 (1.5)	2.8 (1.4)	2.7 (1.4)	0.6	−0.7 (0.3)	−1.2 (0.5)	−0.1 (0.0)
PHQ-9	13.6 (3.6)	9.0 (5.4)	10.4 (4.0)	4.8^†^	−1.8^†^ (0.7)	−1.5 (0.6)	−1.3 (0.5)
IWQOL	54.3 (35.7)	50.7 (32.9)	50.0 (32.8)	1.7	−0.7 (0.3)	−0.7 (0.3)	−0.4 (0.2)
RSES	2.3 (0.7)	2.7 (0.7)	2.8 (0.8)	9.0*	−1.8^†^ (0.7)	−2.0* (0.8)	−1.8^†^ (0.7)

N = 7; BED, binge-eating disorder; BMI, body mass index; EDE-Q, Eating Disorder Examination-Questionnaire (0–6); IWQOL, Impact of Weight on Quality of Life (0–4); MDD, major depressive disorder; PHQ = Patient Health Questionnaire (0–3); RSES = Rosenberg Self-Esteem Scale (0–3); %EWL = percentage of excess weight loss; χ^2^ = Friedman chi-square statistics; *Z* = Wilcoxon Z-statistics; r = effect size (r) interpreted as small ≤ 0.3, medium ≤ 0.5, large ≥ 0.05. **p < 0.01, *p < 0.05, ^†^p < 0.10.

Changes in the frequency of BED and MDD diagnoses were not significant (all *p*s > 0.05), however, small to medium effect sizes indicated decreases in the frequency of diagnoses from pre- to post-treatment (φBED = 0.3; φMDD = 0.4), as well as from pre- and post-treatment to 3-month follow-up (all φs ≥ 0.4). In detail, one patient was diagnosed with BED of low frequency/limited duration with SBEs and one patient fulfilled MDD criteria at post-treatment. At 3-month follow-up, only one patient was diagnosed with a BED of low frequency/limited duration with OBEs and none of the patients fulfilled MDD criteria.

For self-reported eating disorder psychopathology, depressive symptoms, and quality of life no significant changes were found from pre- to post-treatment as well as from pre-treatment to 3-month follow-up. Self-esteem significantly increased from pre-treatment to 3-month follow-up. Effect sizes, however, indicated small to large effect changes: For eating disorder psychopathology, a small effect decrease from pre- to post-treatment and a large effect decrease from pre-treatment to 3-month follow-up was found. From pre- to post-treatment as well as 3-month follow-up, a large effect decrease in depressive symptoms was found. Although there was an increase of depressive symptoms from post-treatment to 3-month follow-up. For quality of life, medium effect changes between pre- and post-treatment as well as pre-treatment and 3-month follow-up indicated a decrease of impact of weight on quality of life. Finally, large effect sizes indicated increasing levels of self-esteem from pre- to post-treatment and from pre-treatment to 3-month follow-up.

## Discussion

We examined feasibility and effects of CBT addressing BED and MDD in patients following bariatric surgery. The POSTBAR manual comprised evidence-based CBT interventions in 15 individual sessions that aimed for improving long-term weight loss outcome, psychopathology, and psychosocial functioning. Recruitment of patients was manageable, acceptance of treatment was high, and retention rates were comparable with those previously published (13, 21). Our results showed significant reductions in body weight and medium to large effect sized improvements in eating disorder psychopathology, depressive symptoms, and self-esteem. More importantly, most of these changes remained stable during 3-month follow-up. Overall, findings extended evidence for the positive effect of behavioral management on weight loss outcome and psychosocial functioning (21).

A major strength of the study is the thorough psychopathological assessment, as BED and MDD were assessed via clinical expert interviews. Additionally, inclusion and exclusion criteria allowed for the recruitment of a diverse sample of RYGB surgery patients with regard to age, body weight, weight loss, and interval since surgery. Thus, the study procedure and the adapted manual are applicable to patients independently of whether they are within their initial weight loss phase or whether they have already gained weight. However, further research should investigate when post-operative CBT should best be delivered.

The results need to be interpreted with regard to several limitations. First, the study likely had insufficient power to detect statistically significant differences. Therefore, we provided effect sizes for interpretation of the non-significant test statistics in the small sample. Second, the significant decrease of body weight should be interpreted with caution. Four of five patients have had surgery less than 2 years before study inclusion and might therefore still have been in their initial weight loss phase. Pre-treatment characteristics, however, documented that half of them already reported weight regain following their lowest post-operative body weight. Nevertheless, following CBT treatment, all of them had lost body weight at post-treatment and 3-month follow-up. Third, given the uncontrolled study design, we could not draw causal interpretation in the way that improvements could be solely attributed to the CBT interventions. Psychosocial changes could also be attributed to patient and setting biases, expectancy and demand characteristics, and time and assessment effects (39).

Further research avenues arise from the results and limitations described above. This feasibility study provides valuable information to adapt the study procedure for proof-of-efficacy in a randomized-controlled trial (RCT) taking the following considerations into account: First, recruitment data suggest that more than one half of the eligible patients were unable to attend weekly therapy sessions due to a lack of time and travel distance. Therefore, new methods such as telephone-based (23) or Internet-based CBT (27) might increase access to treatment for these patients. Moreover, previous studies found post-operative BED and MDD in up to a quarter of the patients, thus, instruments for reliable and valid assessment of mental disorders should be part of routine aftercare. Second, weight loss is consistently reported as primary outcome in trials on post-operative behavioral interventions (21), however, weight loss is also influenced by surgical procedure (e.g. gastric bypass vs. gastric sleeve) and interval since surgery (e.g. before or after honeymoon phase) (40). Therefore, inclusion criteria for an RCT examining the efficacy of CBT on weight loss outcome should be considered carefully. For example, the variety of surgical procedures should be limited to a small number and interval since surgery should be as homogenous as possible. Alternatively, both factors could be controlled for in statistical analysis requiring a larger initial sample size. Third, future studies should not solely focus on weight loss outcome. Due to the fact that CBT interventions aimed at both reducing psychopathology and improving patients' psychosocial adaptations, diverse aspects of psychopathology and psychosocial functioning should be examined using reliable and valid structured clinical interviews and self-report questionnaires. In doing so, efficacy of CBT for post-operative mental disorders could be established reliably.

To conclude, successful management of post-operative BED and MDD resulted in improvements of long-term weight loss outcome following bariatric surgery, as well as lower levels of psychopathology and higher levels of psychosocial functioning. Thus, treatment of post-operative BED and MDD is likely to prevent poor weight loss and weight regain associated with a recurrence of medical comorbidities after initial remission (41, 42) and, consequently, might limit additional costs from continued health care management, revision surgeries, and decreased work productivity (43, 44).

## Data Availability Statement

The datasets for this article are not publicly available due to data safety restrictions. Requests to access the datasets should be directed to the corresponding author.

## Ethics Statement

The studies involving human participants were reviewed and approved by Ethical Committee at the Medical Faculty, Leipzig University (Az. 228-12-02072012). The patients/participants provided their written informed consent to participate in this study.

## Author Contributions

Study conception and design: AR, AH. Data acquisition and analysis: AR. Data interpretation: AR, AH. Drafting the work: AR. Critical revision for important intellectual content: AH. Final approval of the version to be published: AR, AH. Agreement to be accountable for all aspects of the work in ensuring that questions related to the accuracy or integrity of any part of the work are appropriately investigated and resolved: AR, AH.

## Funding

This research was supported by grant 01EO1001 from the Federal Ministry of Education and Research (BMBF) and the Open Access Publishing Fund of the University of Leipzig.

## Conflict of Interest

The authors declare that the research was conducted in the absence of any commercial or financial relationships that could be construed as a potential conflict of interest.
